# Impact of Dehydration Processing on Scallop (*Patinopecten yessoensis*) Adductor Muscle: Structural and Oxidative Insights

**DOI:** 10.3390/foods14060948

**Published:** 2025-03-11

**Authors:** Huaqiong Li, Yulong Zhao, Jian Shi, Manat Chaijan, Xichang Wang, Mingyu Yin

**Affiliations:** 1 College of Food Science and Technology, Shanghai Ocean University, Shanghai 201306, China; lhq18214@163.com (H.L.); zylpower@163.com (Y.Z.); s19895905220@163.com (J.S.); xcwang@shou.edu.cn (X.W.); 2 Shanghai Engineering Research Center of Aquatic-Product Processing and Preservation, Shanghai 201306, China; 3 Food Technology and Innovation Research Center of Excellence, School of Agricultural Technology and Food Industry, Walailak University, Nakhon Si Thammarat 80160, Thailand; cmanat@wu.ac.th

**Keywords:** scallop (*Patinopecten yessoensis*), protein oxidation, structural changes, drying process, seafood quality

## Abstract

This study investigated the impact of four drying techniques—hot air drying (HAD), vacuum hot air drying (VFAD), microwave drying (MWD), and vacuum freeze-drying (VFD)—on the structural, physicochemical, and functional properties of scallop adductor muscles, a critical marine resource in the food industry. The results demonstrated that VFD optimally preserved the ultrastructural integrity of the tissue, maintaining its surface fibrous architecture and achieving a superior recovery ration (78%) and rehydration ration (186.5%) compared to HAD, VFAD, and MWD. While the zeta potential remained statistically invariant across methods, HAD induced the largest particle agglomeration, followed by MWD. Notably, VFD enhanced protein stability, increasing the sulfhydryl content by 163.2% and reducing carbonyl formation by 48.1% relative to HAD, whereas MWD had the opposite effect. Multispectral analyses revealed the severe disruption of protein secondary and tertiary structures after MWD, while VFD minimized conformational denaturation. Statistical modeling ranked the drying sensitivity parameters as follows: surface hydrophobicity > hardness> β-turn content > dityrosine crosslinking > transverse relaxation time T23. These findings underscore VFD as the optimal method for mitigating structural degradation and oxidative damage in scallop processing, providing actionable insights to enhance the technofunctional quality of shelf-stable scallop products.

## 1. Introduction

Scallop (*Patinopecten yessoensis*) is a kind of seafood with a great economic value. In 2023, the total output value of global scallop farming reached USD 8.4 billion [1]. Scallops are rich in protein, unsaturated fatty acids (such as omega-3 fatty acids), vitamins, and minerals, which are beneficial to human health [2]. The adductor muscle of live scallops has a high moisture content (ranging from 76.5% to 85.5%), which makes it prone to spoilage and decay due to the growth of bacteria, molds, and other microorganisms [3,4]. Under ambient conditions (20–35 °C), fresh scallops may only be preserved for 1 to 2 days, while dried scallops can be stored for months or even years [5]. This facilitates the supply of scallops in the off-season [2], which are small in size and light in weight. Compared with fresh scallops, it takes up less space and does not require sophisticated cold-chain transportation equipment, reducing transportation costs and storage difficulties and expanding their sales range [6]. Dried scallops could be used as a unique ingredient in cooking. The drying process of scallops is an important research issue.

Drying processes fall into two categories: thermal drying and non-thermal drying. Moisture can be removed from seafood through boiling and drying [7]. During the drying process, the physical and chemical properties, nutritional components, and flavor of seafood would change significantly [1,6,8]. While natural drying (ND) at room temperature, salt drying, and hot air drying (HAD) are the most widely used low-cost seafood drying techniques, it is quite challenging to obtain high-quality dried food [9]. Many novel high-quality drying techniques have been applied to seafood [10], including microwave drying (MWD), vacuum freeze-drying (VFD), and combined drying [11]. These techniques retain the original flavor compounds and nutritional components of seafood [6]. HAD increases the muscle density while causing the severe denaturation and degradation of the secondary structure of proteins, and VFD technology could produce a porous final product [12]. It was found that the MWD of *Gastrodia elata* effectively reduced the drying time and energy consumption [8,9]. VFD can promote the flavor release of whitebait [8], mushrooms [7], tilapia fillets [10], and scallops muscle [6], thus increasing the desire for consumption.

Aquatic proteins possess numerous crucial nutritional functions [13]. It is an excellent source of essential amino acids (i.e., leucine and lysine), and the amino acid composition ratio is close to the human body’s requirements [14]. In meat products, the spatial structure of proteins determines the texture of the food, such as the tenderness [15]. The degree of protein denaturation and oxidative degradation is related to environmental factors such as the temperature, humidity, and oxygen during the drying process [12]. The stability of the scallop adductor muscle depends on the spatial network structure of proteins [2]. During the drying process, myofibrillar proteins would undergo denaturation and oxidation, which are manifested as the breakage of protein backbones, cross-linking between proteins, and modifications to amino acid side chains [8,9]. It is confirmed that dehydration processing promoted the shrinkage of cell structures and browning [8,9]. The secondary structure of proteins is particularly sensitive to heat treatment and is significantly correlated with hardness [9]. Tandem mass tag (TMT) proteomics reveal that the molecular weight of shrimp tail proteins is associated with the textural quality [12]. Thus, it is of great significance to study the effects of different drying methods on protein oxidation and scallop adductor muscle quality.

This study is aimed at comparing the protein structures, texture profile, and physicochemical properties of dried scallop adductor muscles (DSAM) treated with HAD, vacuum hot air drying (VHAD), MWD, and VFD techniques. Additionally, this study aims to investigate the protective mechanisms and functional preservation of scallop myofibrillar proteins under the optimal drying method during the drying process. We hypothesize that vacuum freeze-drying (VFD) will better preserve protein integrity compared to conventional hot air drying (HAD) and microwave drying (MWD).

## 2. Materials and Methods

### 2.1. Materials

Live scallops (weight = 122.79 ± 15.98 g, *n* = 100) were purchased from Luchaogang seafood market in Shanghai and transported to the lab in an icebox within 4 h. The sodium chloride, 2.5% glutaraldehyde fixative, and disodium hydrogen phosphate were purchased from McLean Biochemical Science and Technology Co., Ltd. (Shanghai, China). The other reagents involved in this work were purchased from Yuanye Biotechnology Co., Ltd. (Shanghai, China).

### 2.2. Drying Process

The fresh adductor muscles (moisture content: 76.89 ± 3.14%) were treated according to the previously established methods [6]. Briefly, the samples were dissected from the shell and cut into cubes (SPC, 20 mm × 20 mm × 15 mm) on ice. The samples were weighed and dried using various methods until achieving moisture content ≤ 12%. HAD group sample was treated at 50 °C for 690 min, using hot-air oven (DGG-9203AD, Senxin Experimental Instrument Co., Ltd., Shanghai, China). VHAD group sample was treated at 50 °C for 600 min, using vacuum hot-air oven (VC50, Salvis, Switzerland). MWD group sample was treated for 35 min at 80 W power, using microwave oven (EG823MF7-NRH3, Midea, Guangdong, China). VFD group sample was treated at for 372 min, using freeze-dryer (BOC EDWARDS, Shiyou Venture Technology Co., Ltd., Beijing, China).

### 2.3. Moisture, Water Activity (Aw), and Rehydration and Recovery Ratio Measures

The moisture content of DSAM was determined according to the Association of Official Analytical Chemists (AOAC). The Aw was investigated by a water activity meter (CR-10, Konica Minolta, Tokyo, Japan). The test was performed 3 times.

The DSAM was immersed in a 40 °C water bath for 30 min; the rehydration ratio was calculated as followed:

(1) $$R_{reh}=\frac{m-m_{t}}{m_{t}}$$

(2) $$R_{rec}=\frac{m}{m_{1}}$$

Here, R reh is the rehydration ratio of the sample, m is the post-rehydration weight, m_t_ is the pre-rehydration weight, R rec is the recovery ratio (%) of SPC, and m_1_ is the weight (g) of fresh SPC before drying.

### 2.4. Low-Field Nuclear Magnetic Resonance (LF-NMR) Measurement

The LF-NMR analysis was performed as described in reference [16]. The samples were cut into cylinders with a height of 20 mm, wrapped in plastic wrap, and placed in an NMR tube. The sample signals were acquired by Carr–Purcell–Meiboom–Gill (CPMG) pulse sequence and using the following parameters: proton resonance frequency (SF) of 21 MHz, receiver bandwidth (SW) of 200 kHz, control parameter for the start time of sampling (RFD) of 0.08 ms, analog gain (RG1) of 10 dB, digital gain (DRG1) 3, and preamplification gain (PRG) of 1.

### 2.5. Microstructural Measurements

The samples were fractured and coated with gold powder; the cross-section was observed with a scanning electron microscope (SEM) (S–3000N, Hitachi, Tokyo, Japan) [17].

### 2.6. Color

The color was investigated by a chromometer (CR-10, Konica Minolta, Japan) with the measurement of L, a*, and b*. L represents lightness, with higher values indicating lighter hues and lower values indicating darker hues. The a* and b* values describe color biases: a* indicates the red–green axis (positive values represent a red bias, negative values a green bias), and b* indicates the yellow–blue axis (positive values represent a yellow bias, negative values a blue bias).

Based on the obtained color coordinates, values for chroma (c) and whiteness (WH) of the dried sample were computed in cylindrical coordinates [18]:

(3) $$c=\sqrt{{\left(a*\right)}^{2}+{\left(b*\right)}^{2}}$$

(4) $$WH=100-\sqrt{{\left(100-L\right)}^{2}-{\left(a*\right)}^{2}-{\left(a*\right)}^{2}}$$

### 2.7. Texture Profile

The samples’ texture profiles were measured using a texture analyzer (TA-XT Plus, TA XT Plus, Godalming, UK). And the test was performed with 50% sample deformation at a test speed of 0.5 mm/s using a flat-bottom cylindrical probe (6 mm in diameter).

### 2.8. Structural Properties and Oxidation Properties of Dried SPC Myofibrillar Proteins (MP)

#### 2.8.1. MP Extraction

The extraction of protein was referring to the method [19] with minor modification. The DSAM was added in 10 mL ice water and homogenized for 1 min, using homogenizer (FJ200-SH, Shanghai Taxidermy Model Factory, Shanghai, China), and stirred at low speed for 30 min at 4 °C. The homogenate was centrifuged and the supernatant was collected. To the precipitate, 10 mL Tris-HCl buffer (50 mmol/L KCl, 20 mmol/L Tris–HCl, and 1.0 mmol/L EDTA pH 7.0) was added. The precipitate was added into 10 mL buffer (0.6 mol/L KCl, 20 mmol/L Tris-HCl, and pH 7.0), homogenized for 1 min, allowed to stand for 12 h at 4 °C, and then centrifuged at 10,000 r/min for 15 min. The supernatant was obtained as salt-soluble myofibrillar protein (MP) solution. Their contents were determined by the Biuret method [20].

#### 2.8.2. Determining Protein Total Sulfhydryl, Carbonyl Content, and Solubility

The total sulfhydryl and carbonyl content of MP was determined using sulfhydryl (A087-1, Jiancheng Bioengineering Research Institute, Nanjing, China) and carbonyl kits (A063-1, Jiancheng Bioengineering Research Institute, Nanjing, China). The solubility was determined using the method described in reference [21].

#### 2.8.3. Determining Dityrosine Content

MP dityrosine content was quantified using the fluorescence spectrophotometer (F-7100 FL Spectrophotometer, Hitachi, Tokyo, Japan). The MP solution was diluted to 0.5 mg/mL of protein with 50 mmol/L phosphate buffer (pH 7.50, 0.6 mol/L NaCl). Fluorescence intensity was measured using an excitation wavelength of 325 nm and an emission wavelength of 420 nm [22].

#### 2.8.4. Determining Tryptophan Intrinsic Fluorescence

Sample homogenates were prepared following the previously described tryptophan intrinsic fluorescence analysis procedure. The final concentration of MP was 0.1 mg/mL. The emission spectra were recorded from 300 to 400 nm with an excitation wavelength of 295 nm (F-7100 FL Spectrophotometer, Hitachi, Tokyo, Japan) [20].

#### 2.8.5. Determining Surface Hydrophobicity

Surface hydrophobicity was measured using the bromophenol blue (BPB) (Merck, Shanghai, China) method [23]. The amount of bound BPB per mg of protein was calculated by:

(5) $$BPB\;bound\left(\mu g\right)/mg\;protein=200\;\mu g\;BPB\times \left[\frac{A_{control}-A_{sample}}{A_{control}}\right]$$

Note: A is the absorption value of the sample at 595 nm.

#### 2.8.6. Ultraviolet-Visible (UV) Absorption Spectrum

The MP was diluted to 1.0 mg/mL with 20 mmol/L PBS buffer (pH 7.50, 0.6 mol/L NaCl). After filtrating with filter paper, the UV absorption spectra of the diluted solution were scanned at 240–320 nm with a spectrophotometer (UV–1800PC, Mapada, Shanghai, China). PBS buffer (pH 7.50, 0.6 mol/L NaCl) was used as a control [24].

#### 2.8.7. Sodium Dodecyl Sulfate–Polyacrylamide Gel Electrophoresis (SDS-PAGE)

Electrophoresis was performed in a 5% stacking gel and 12% separating gel. A total of 12 μg of protein was loaded onto the sample well in the stacking gel and the sample was subjected to electrophoresis at 80 V for 30 min, followed by 120 V for 90 min. After separation, the gels were stained with 0.1% Coomassie brilliant blue R-250 (Yeasen Biotechnology Co., Ltd., Shanghai, China) and detained with a solution containing 45% methanol and 9% acetic acid [25].

#### 2.8.8. Particle Size and Zeta Potential

The 0.1 mg/mL MP solution (1.0 mL) was added to the Zetasizer Nano ZEN3600 (Malvern Panalytical Ltd., Malvern, UK) cuvette for particle size and zeta potential measurement [24]. The data are presented as particle mean diameter (nm) and zeta potential (mV).

### 2.9. FT-IR Analysis of Myofibrillar Proteins

The MP was freeze-dried for 24 h and each sample (0.5−1.5 mg) was blended with KBr (120 mg) into fine powder and pressed into a tablet. The samples’ FT-IR spectra were scanned at room temperature (25 °C) using an FT-IR spectrometer (Spotlight 400, PerkinElmer, Seer Green, UK). Each spectrum was recorded with 16 scans and 4 cm^−1^ resolution in the 4000−600 cm^−1^ range. The proteins’ secondary structures were calculated using Gaussian distribution fitting of 1600–1700 cm^−1^ using Peak-fit software 5.0 (Systat Software Inc., San Jose, CA, USA) [26].

### 2.10. Statistical Analysis

Results are shown as mean values ± standard deviation. SPSS 21.0 (Chicago, IL, USA) was used for statistical analysis. The data were analyzed by one-way analysis of variance (ANOVA). *p* < 0.05 was considered statistically significant. All measurements were conducted in triplicate, and experiments were independently repeated three times.

## 3. Results and Discussion

### 3.1. Rehydration Ratio, Recovery Ration, and Aw

**Figure 1:**
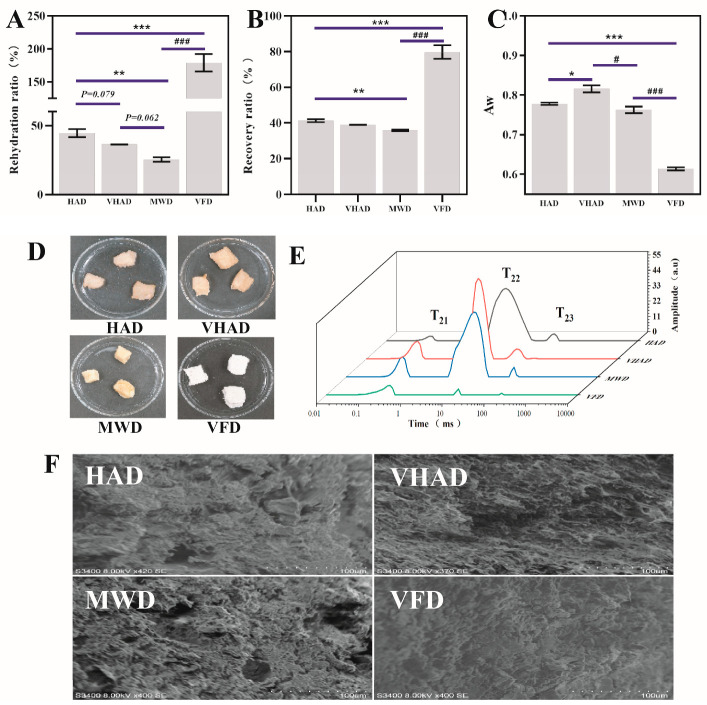
Effects of different drying methods on (**A**) rehydration ratio, (**B**) recovery rate, (**C**) water activity, (**D**) appearance, (**E**) moisture distribution, and (**F**) microstructure of scallop *Patinopecten yessoensis*. Abbreviation: HAD, hot air drying group; VHAD, vacuum hot air drying group; MWD, microwave drying group; VFD, vacuum freeze-drying group; Aw, water activity. The significant difference at *p* < 0.05 determined by ANOVA (Dun-can’s test). * *p* or # *p* < 0.05. ** *p* < 0.01, *** *p* or ### *p* < 0.001 represent significant difference compared to groups determined by Student’s *t*-test.

The rehydration ratio refers to the dried product that could regain its fresh state after water reabsorption [27]. As shown in Figure 1, the rehydration ratio of the VFD group (186.5%) was significantly higher than that in HAD (42.5%) (*p* < 0.05), which might be attributed to the direct sublimation of water from the material during drying. During vacuum freeze-drying, moisture is removed through sublimation (direct transition from ice to vapor) rather than liquid evaporation. This approach avoids the structural damage caused by the liquid water movement, preserving the material’s original porous structure and fiber integrity. Such an intact structure enhances water absorption during rehydration [28]. Furthermore, the material’s structural integrity was maintained, and the sample exhibited a more robust water absorption capability, increasing the rehydration ratio [29]. The hydration ratio of the HAD group was higher than that of the VHAD (*p* = 0.079) and MWD group (*p* < 0.01). There was no significant difference between the MWD (27.6%) and VFAD (36.4%) rehydration rates (*p* = 0.062). MWD dries quickly but with rapid internal water evaporation, leading to temperature differentials that could harden or crack the surface.

The recovery ratio refers to the proportion of the active ingredients or mass retained from the raw materials after drying [4]. A high rehydration rate means that the food can restore its original shape and texture after reabsorbing water, which usually indicates that the structure of the food has been preserved during the drying process [9]. The VFD recovery rate was found to be the highest and around 40% for HAD, VHAD, and MWD (Figure 1B). The water activity level has far-reaching significance for multiple links such as food processing, storage, and quality control. The VFD Aw (0.607~0.611) was significantly lower than VHAD (*p* < 0.001). The MWD has an Aw value arranged 0.785~0.792. There was no significant difference in the water activity values of HAD and MWD (*p* > 0.05). The Aw of 0.60 is often the minimum threshold for microbial growth, making the food less likely to spoil over the ensuing two years [30]. Therefore, scallops dried with VFD exhibit higher rehydration and recovery rates compared to HAD, VHAD, and MWD.

### 3.2. Distribution and Mobility of Water

LF-NMR could be used to analyze the state, distribution, and quantity of water using T2 relaxation times and the area of the A2 peak [31]. The water states included bound (T_21_, 1–10 ms), immobilized (T_22_, 10–100 ms), and free water (T_23_, 100–1000 ms); immobilized water is the most common [32]. The T_2_ peak area and transverse percent relaxation times were shown in Figure 1E. Compared to HAD, the T_22_ peak in MWD shifted to the left, the peak area decreased, and the transverse relaxation times were shortened. Among the four drying methods, VFD showed the smallest T_22_ peak area, indicating that the VFD process results in the least amount of water movement within the scallop slices, leading to the most pronounced contraction of the scallops’ muscle fibers [27]. It was suggested that the tighter the network structure, the stronger the water binding and the shorter the T2 [33]. Compared to HAD, VHAD, and MWD, VFD significantly reduced the A22 peak area, indicating that VFD facilitates the conversion of immobilized to free water, which evaporates during the drying process [27].

### 3.3. SEM Observation

The ultrastructural observation of muscle fibers typically requires an electron microscope to reveal the cell’s fine interior details [34]. The SEM of DSAM is shown in Figure 1F. Compared to HAD, VHAD, and MWD, VFD resulted in a more intact structure; the other drying methods resulted in larger holes in the MP and uneven fiber gaps. HAD primarily relies on external hot air for moisture removal; this might cause rapid moisture evaporation from the scallop’s surface without improving the uneven internal moisture distribution, leading to shrinkage and microstructural deformation [30,35]. Under vacuum conditions, the drying temperature is lowered, allowing for uniform moisture evaporation and reducing issues caused by surface hardening and uneven internal moisture distribution [4,36]. Therefore, VHAD might better preserve the scallop’s microstructural integrity than HAD, reducing fiber shrinkage and deformation.

### 3.4. Color and Texture Profile of DSAM

Color is a visual metric indicator of a food product, with L, a*, b*, and c values reflecting its lightness, red–blue, yellow–green, and combined chromaticity values, respectively [37]. The significantly greater L*, a*, and whiteness values observed in the VFD-treated DSAM compared to HAD, VHAD, and MWD groups correlated with the visual changes in the product appearance, as demonstrated in Figure 1B and Table 1. During the drying process, the Maillard reaction and the caramelization reaction intensify [9]. The sugars and amino acids on the surface react under the influence of smoke and a high temperature [36], and the color gradually changes to a reddish-brown or even a dark brown. The texture of aquatic products is of great significance in aspects such as evaluating the quality, freshness, processing suitability, and consumer acceptance of aquatic products. The Firmness of DSAM was in the order HAD > VHAD > MWD > VFD, and the HAD treatment was significantly higher than that in VHAD, MWD, and VHAD (*p* < 0.05), while the hardness of DSAM was in the order: VHAD > MWD > HAD > VFD. Moisture loss during drying leads to structural shrinkage, which increases the firmness and hardness, making the product denser and chewier. These findings align with previous studies on *Penaeus vannamei* shrimp [12], beef [36], and mushrooms [9], which also reported increased firmness and hardness following drying.

### 3.5. Solubility, Turbidity, and Surface Hydrophobicity of MP

**Figure 2:**
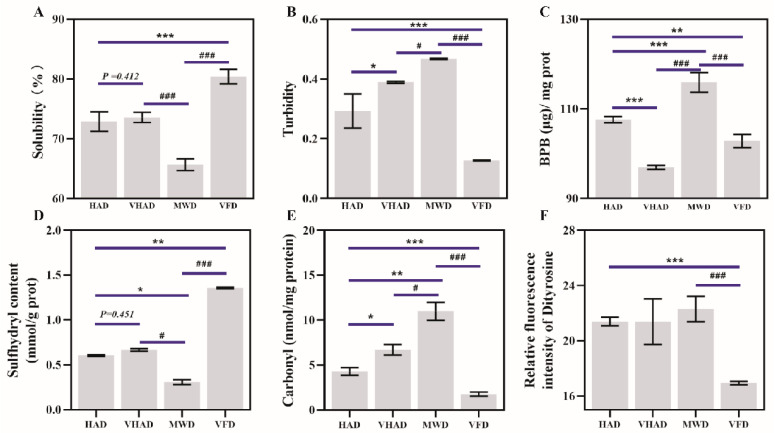
Effects of different drying methods on (**A**) solubility, (**B**) turbidity, (**C**) surface hydrophobic properties, (**D**) sulfhydryl content, (**E**) carbonyl content, and (**F**) dityrosine of scallop *Patinopecten yessoensis*. Abbreviation: HAD, hot air drying group; VHAD, vacuum hot air drying group; MWD, microwave drying group; VFD, vacuum freeze-drying group. The significant difference at *p* < 0.05 determined by ANOVA (Duncan’s test). * *p* or # *p* < 0.05. ** *p* < 0.01, *** *p* or ### *p* < 0.001 represent significant difference compared to groups determined by Student’s *t*-test.

As depicted in Figure 2A, there was a significant difference in the solubility between VFD and SF (*p* < 0.05). Solubility was 77.25 ± 2.64% for fresh samples and then increased to 81.68 ± 3.81% in the VFD group. Under high-temperature conditions, proteins undergo thermal denaturation, causing the peptide chain to unfold and exposing numerous hydrophobic groups on the protein’s surface. Driven by hydrophobic interactions, protein aggregation reduces solubility. For example, corn protein’s solubility decreases with the increasing drying temperature [38].

The turbidity of a protein solution depends on the solute particle size and turbidity is commonly used to characterize the degree of protein aggregation and dissociation [39]. Turbidity after HAD and VHAD treatments was significantly higher than the VFP group (*p* < 0.05). The turbidity under MWD was significantly higher than the HAD and VHAD group (*p* < 0.05). This was attributed to the influence of nonthermal effects, such as electromagnetic fields, during short-term microwave treatment, which disrupt noncovalent bonds that maintain the protein spatial structure [4,36].

The MP surface hydrophobicity refers to the weak affinity of surfaces for water or other polar liquids, usually related to the degree of exposure of hydrophobic groups on the protein surface [34]. After VHAD, the surface hydrophobicity was lower than the HAD and MWD samples (*p* < 0.05). There was no significance in the VHAD and VFP group. Moderate oxidative modifications that open disulfide bonds exposed the hydrophobic residues of MFP, thereby increasing the surface hydrophobicity [34]. The process also led to the cross-linking and aggregation of disulfide bonds in MP, which conceals hydrophobic groups and reduces surface hydrophobicity [40]. Disrupting a protein’s hydrophobic domains reveals aromatic residues, enhancing the fluorescence emission [41]. These results were consistent with the inherent tryptophan fluorescence intensity. The thiol content and fluorescence intensity changes reflect structural damage within the dried scallop MP. The disruption of the protein structure affects the capacity of MP to hold water, which is detrimental to the subsequent DSAM storage.

### 3.6. Sulfhydryl and Carbonyl Content of MP

Total sulfhydryl groups are the sum of a sample’s sulfhydryl (-SH) groups. Cysteine is susceptible to attack by reactive oxygen species, which could convert sulfhydryl groups to disulfide bonds, resulting in changes in the sulfhydryl content [42]. The drying method significantly impacts the total sulfhydryl content of DSAM proteins. As shown in Figure 2D, the sulfhydryl content of HAD is significantly reduced compared to VFD (*p* < 0.05). There is a significant difference between MWD and VFD in the sulfhydryl content (*p* < 0.05). The high-temperature environment promotes disulfide bonding and protein aggregation during HAD [43]. It was previously reported that thermal stress affects the mitochondrial function, accumulating free radicals and MP structural changes [44]. Furthermore, VFD treatment could shorten the drying time, which prevents the further oxidation of the proteins compared to the other three drying methods.

The carbonyl group is an organic functional group (C=O) consisting of carbon and oxygen atoms, connected by a double bond [45]. The carbonyl content of the MWD was 10.4 ± 1.52 nmol/mg protein, while the carbonyl content of the VFD was 1.81 ± 0.19 nmol/mg protein. The carbonyl content after MWD exhibits significant differences from the other samples (*p* < 0.05). Oxidation accelerates at higher temperatures, leading to peptide chain breakage and the oxidation of free amino and amino groups to carbonyls, accumulating carbonyl [44]. VFD is performed under negative pressure and at lower temperatures, which safeguards proteins from radicals’ harmful effects and reduces the oxidation reaction rate [6]. These factors preserved the proteins’ integrity and activities [46].

### 3.7. Dityrosine and Tertiary Structure of MP

**Figure 3:**
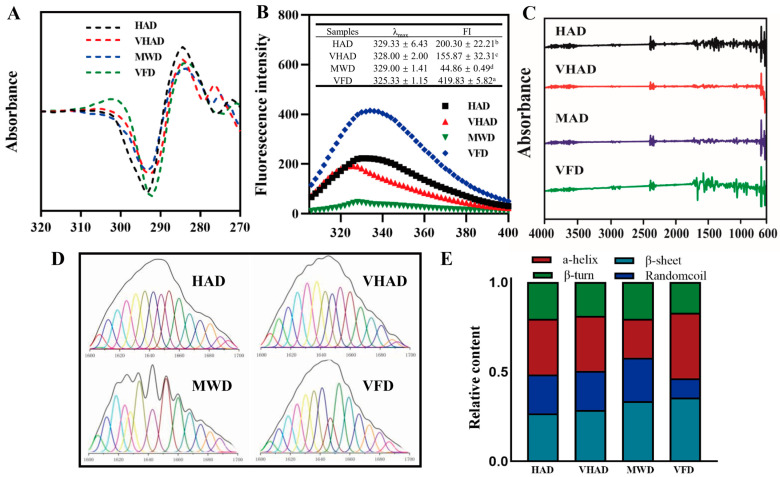
Effects of different drying methods on (**A**) second-order derivative mapping of UV absorption spectra, (**B**) fluorescence spectrum, (**C**) infrared spectrum, (**D**) infrared second-order derivative fitting curve, and (**E**) relative content of protein secondary structure of scallop *Patinopecten yessoensis* myofibrillar protein. Abbreviation: HAD, hot air drying group; VHAD, vacuum hot air drying group; MWD, microwave drying group; VFD, vacuum freeze-drying group. The significant difference at *p* < 0.05 determined by ANOVA (Duncan’s test). ^a,b,c,d^ Means in the same column with different superscripts differ significantly (*p* < 0.05).

Dityrosine’s relative fluorescence intensity is the numerical value of the fluorescence signal intensity produced by dityrosine [47]. After undergoing VFD treatment, the dityrosine content decreased to 16.8%. It was illustrated that the relative fluorescence intensity of VFD was significantly lower than HAD, VHAD, and MWD (*p* < 0.001). The fluorescence intensity of tryptophan residues expresses the tertiary structure of proteins [40]. As shown in Figure 3, VFD exhibits the highest fluorescence intensity at 325.3 nm, with an intensity of 419.8. HAD, VHAD, and MWD showed sequentially decreased fluorescence intensities. The maximum emission wavelengths for MP after HAD, VHAD, and MWD are 329 nm, 328 nm, and 329 nm, respectively, while VFD is 325 nm, indicating a red shift compared to the other drying methods. This suggested that drying alters the microenvironmental conformation of MP. The drying treatment could change a protein’s structure, affecting its fluorescence properties [9]. In contrast, low-temperature freezing helps preserve the original structure of proteins, thereby maintaining their fluorescence intensity.

### 3.8. Secondary Structure of MP

The secondary structure of a protein refers to the conformation formed by the polypeptide backbone atoms coiling or folding in a specific way along a certain axis. Hydrogen bonds mainly maintain this structure, and its principal forms include an α-helix, β-sheet, β-turn, and random coils [48]. The α-helix content in MWD is noticeably reduced compared to VFD. The stability of the α-helix, related to intramolecular hydrogen bonds, is compromised due to protein oxidation, leading to bond breakage and the transformation of the α-helix into a β-turn [49]. Compared to VFD, HAD samples exhibited reduced β-sheets and increased random coils. These changes indicate that the interaction between proteins and large polymer molecules has been enhanced and, subsequently, the structure properties of myofibrillar proteins have changed [50]. The increase in the β-sheet content is due to the aggregation of proteins caused by interactions between sulfhydryl and hydrophobic groups, forming disulfide bonds, accompanied by transitions between β-turns and β-sheets [51].

### 3.9. SDS-PAGE, Zeta Potential, and Particle Size

**Figure 4:**
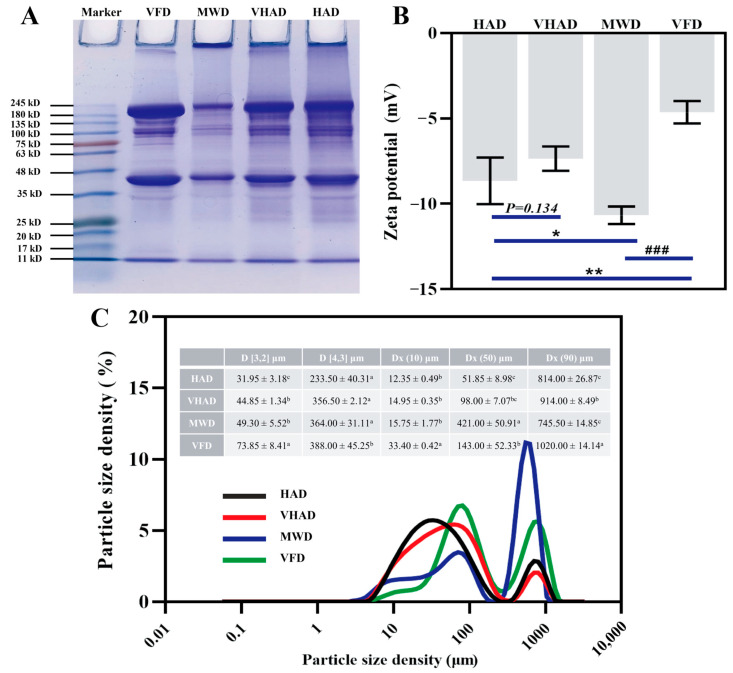
Effects of different drying methods on (**A**) protein molecular weight, (**B**) zeta potential, and (**C**) particle size distribution of scallop *Patinopecten yessoensis* myofibrillar protein. Abbreviation: HAD, hot air drying group; VHAD, vacuum hot air drying group; MWD, microwave drying group; VFD, vacuum freeze-drying group. The significant difference at *p* < 0.05 determined by ANOVA (Duncan’s test). * *p* < 0.05. ** *p* < 0.01, ### *p* < 0.001 represent significant difference compared to groups determined by Student’s *t*-test. ^a,b,c^ Means in the same column with different superscripts differ significantly (*p* < 0.05).

The principle of SDS-PAGE is based on a protein migration rate within an electric field; it is inversely proportional to its molecular weight [52]. As shown in Figure 4A, the myosin heavy chain (MHC, 224 kDa) and actin (43 kDa) were significantly lower in MWD than the other samples, accompanied by larger protein molecular interception in the injection port. The MHC and actin were wider in VFD compared to HAD. It was implied that VFD prevents MP degradation [53]. The reduced intensity of MHC and actin protein in the samples might be attributable to protein oxidation during high-temperature drying, resulting in disulfide bond formation and the subsequent aggregation of high-molecular-weight substances [17]. These aggregates decreased the solubility or extractability of MHC, thereby reducing the intensity of the protein bands. Overall, generating free radicals during high-temperature drying processes leads to varying degrees of oxidation in DSAM.

The particle size and zeta potential define the distribution of ions and charge states in an ionic solution. HAD and MWD elevate temperatures, rendering proteins more susceptible to oxidative decomposition than lower temperatures [54]. The particle size and potential of the MWD group were significantly higher than those of HAD, VHAD, and VFD (*p* < 0.05). The HAD group was subjected to drying at increased temperatures under atmospheric pressure, while microwave heating ensured more uniform heat distribution across the sample [55]; this process concentrated the material particle sizes into a smaller range, as evidenced by the much lower difference between DX(50) and DX(90) than in the HAD and VHAD groups (*p* < 0.05).

### 3.10. Correlation Analysis

**Figure 5:**
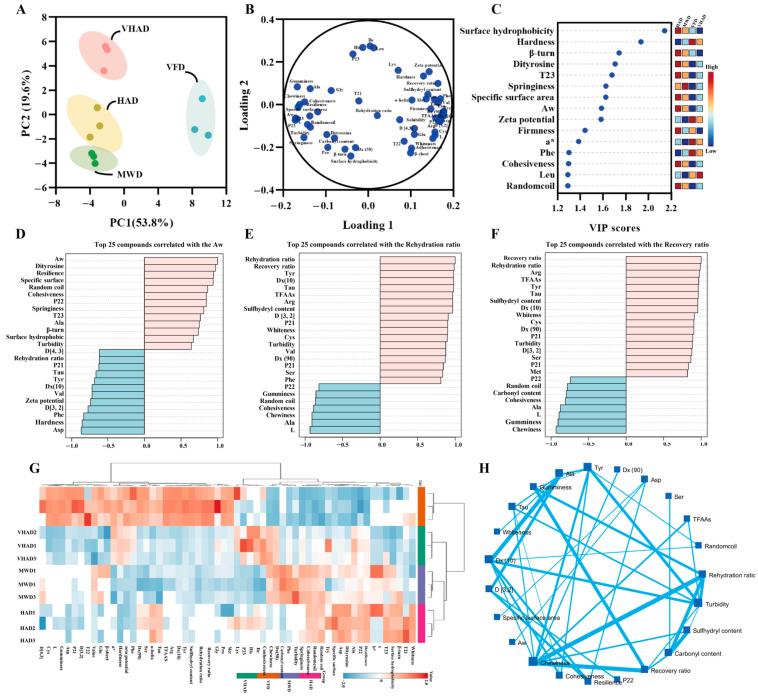
Structural and oxidation properties of scallop (*Patinopecten yessoensis*) adductor muscle. (**A**) Principal component analysis; (**B**) Loading plot; (**C**) VIP value; (**D**) Top 25 compounds correlated with the Aw; (**E**) Top 25 compounds correlated with the Rehydration ratio; (**F**) Top 25 compounds correlated with the Recovery ratio; (**G**) Correlation heat map; and (**H**) Spatial network correlation. (Abbreviation: HAD, hot air drying group; VHAD, vacuum hot air drying group; MWD, microwave drying group; VFD, vacuum freeze-drying group; glycine, Gly; arginine, Arg; leucine, Leu; valine, Val; alanine, Ala; serine, Ser; proline, Pro; isoleucine, Ile; phenylalanine, Phe; lysine, Lys; threonine, Thr; methionine, Met; histidine, His; tyrosine acid, Tyr; aspartic acid, Asp; cysteine, Cys; glutamic acid, Glu).

Factor-based multivariate statistical analysis can achieve the screening of sensitive indicators, such as principal component analysis and correlation cluster analysis [6]. The factor analyses of the test sample metrics are displayed. Principal component factors 1 and 2 were 53.8% and 19.6% (Figure 5), respectively, suggesting that the samples could be interpreted. The HAD, VHAD, and MWD group were closer together, while the VFD group was alone in one and four quadrants. Based on the Loading analysis, it was found that the physicochemical properties and protein structure-related indexes showed positive and negative distributions on Loading 1. The VIP value (>1.0) analysis showed that the surface hydrophobicity, hardness, β-turn, dityrosine, T23, springiness, etc., was sensitive to the drying process. The top 25 compounds correlated with the Aw, rehydration ratio, and recovery ratio analysis showed that all of them are positively correlated with turbidity.

Cluster analysis can be downscaled to achieve similar combinations of features [6]. It was found that the different drying treatments are divided into two large groups, the VFD group, HAD with MWD and VHAD in one group (this is consistent with the results of the PCA analysis), and, in particular, HAD with MWD in the branch. It was illustrated that chewiness was negatively correlated with the recovery ratio (R = −0.923, *p* < 0.001) and rehydration (R = −0.924, *p* < 0.001). Gumminess was positively related to Ala (R = 0.902, *p* < 0.001) and Gly (R = 0.811, *p* = 0.002), while negatively related to Arg (R = −0.965, *p* < 0.001). The carbonyl content was negatively related to the rehydration ratio (R = −0.987, *p* < 0.001) and recovery ratio (R = −0.982, *p* < 0.001), while positively related to Pro (R = −0.979, *p* < 0.001).

Proteins are oxidized during drying, which affects their secondary and tertiary structures and changes the protein’s physicochemical properties [56]. Following the VFD treatment, the scallop exhibits a marked reduction in hardness and chewiness while acquiring a more vibrant, pristine white hue with a subtle reddish tint (Figure 5). In addition, VFD retained a more complete microstructure than HAD, VHAD, and MWD. The other drying methods resulted in larger pores and inhomogeneous fiber gaps in the myofibrillar proteins. The post-VFD scallops looked the best and had the most complete muscle structure. After MWD treatment, the α-helix of MP was converted to β-turn. HAD reduced β-sheets and increased random coils. After HAD treatment, the MP solubility and particle size decreased and hydrophobicity increased. Thus, the HAD proteins were more easily oxidized at higher temperatures. VFD could change its tertiary structure significantly compared to the other drying methods, but the MWD was significantly altered. Compared with the other three groups, the carbonyl content of VFD decreased and the sulfhydryl content increased, indicating that VFD was more capable of maintaining MP stability.

## 4. Conclusions

This study investigated the physicochemical properties and protein oxidation of DSAM treated with different drying methods (HAD, HVAD, MWD, and VFD). MWD was the fastest method for water removal, while VFD exhibited the best rehydration characteristics and the least protein oxidation. Thermal drying techniques (HAD and MWD) resulted in weaker rehydration properties and harder textures, with MWD-treated samples showing the highest surface hardness. All drying methods accelerated protein oxidation, leading to complex spatial structures, with VFD showing the least oxidative damage.

These findings provide a theoretical basis for improving the quality of dried scallop products and selecting appropriate drying methods. Importantly, this study highlights the trade-offs between the drying efficiency, product quality, and protein oxidation, which are critical for industrial applications. Future research could explore the optimization of hybrid drying methods (e.g., combining VFD with thermal techniques) to balance the drying speed and product quality. Additionally, further investigation into the molecular mechanisms underlying protein oxidation during drying and its impact on nutritional and sensory properties could offer valuable insights for the development of high-quality dried seafood products.

## Figures and Tables

**Table 1:** Texture and color of scallop *Patinopecten yessoensis* induced by different dehydration processing.

Index	HAD	VHAD	MWD	VFD
Firmness	3820.31 ± 99.16 ^a^	3140.14 ± 315.37 ^ab^	2867.98 ± 598.14 ^b^	2104.95 ± 422.80 ^c^
Hardness (g)	1893.49 ± 58.47 ^c^	3500.72 ± 61.05 ^a^	2553.39 ± 5.33 ^b^	1204.95 ± 116.67 ^d^
Adhesiveness (g/s)	−22.24 ± 0.51	−37.96 ± 7.1	−13.03 ± 0.86	−9.05 ± 1.23
Springiness	0.69 ± 0.03 ^b^	0.63 ± 0.01 ^c^	0.84 ± 0.00 ^a^	0.42 ± 0.03 ^d^
Cohesiveness	0.58 ± 0.01 ^b^	0.57 ± 0.01 ^b^	0.67 ± 0.01 ^a^	0.24 ± 0.03 ^c^
Gumminess	1041.65 ± 42.94 ^c^	2103.18 ± 91.26 ^a^	1770.37 ± 29.52 ^b^	820.19 ± 65.75 ^d^
Chewiness	699.28 ± 16.12 ^b^	1380.26 ± 21.60 ^a^	1392.87 ± 89.67 ^a^	368.56 ± 43.21 ^c^
Resilience	0.20 ± 0.01 ^a^	0.19 ± 0.00 ^b^	0.17 ± 0.00 ^c^	0.07 ± 0.01 ^d^
L	50.82 ± 3.05 ^d^	52.22 ± 1.23 ^d^	67.53 ± 3.54 ^b^	87.85 ± 2.63 ^a^
a*	−1.04 ± 0.44 ^c^	0.87 ± 0.73 ^a^	−3.09 ± 0.46 ^d^	1.21 ± 0.42 ^a^
b*	14.74 ± 4.53 ^b^	14.73 ± 0.96 ^b^	20.19 ± 3.72 ^a^	13.13 ± 2.55 ^b^
Whiteness	48.45 ± 2.42 ^d^	49.98 ± 1.22 ^d^	61.48 ± 3.45 ^b^	81.89 ± 2.47 ^a^
c	14.78 ± 4.53 ^b^	14.77 ± 0.96 ^b^	20.43 ± 3.69 ^a^	13.19 ± 2.55 ^b^

^a,b,c,d^ Means in the same column with different superscripts differ significantly (*p* < 0.05).

## Data Availability

The original contributions presented in this study are included in the article; further inquiries can be directed to the corresponding author.
